# Engagement of Adolescents in a Health Communications Program to Prevent Noncommunicable Diseases: *Multiplicadores Jóvenes*, Lima, Peru, 2011

**DOI:** 10.5888/pcd12.140416

**Published:** 2015-03-05

**Authors:** Francisco Diez-Canseco, Yulissa Boeren, Renato Quispe, Mey lin Chiang, J. Jaime Miranda

**Affiliations:** Author Affiliations: Francisco Diez-Canseco, Yulissa Boeren, Renato Quispe, CRONICAS Center of Excellence in Chronic Diseases, Universidad Peruana Cayetano Heredia, Lima, Peru; Mey lin Chiang, School of Communication, Universidad de Lima, Lima, Peru.

## Abstract

**Background:**

Several risk factors for noncommunicable diseases (NCDs), including obesity, are associated with behaviors established in infancy that persist throughout adolescence and adulthood. As such, adolescents should be engaged in the design and implementation of NCD prevention strategies.

**Community Context:**

In Lima, Peru’s capital, the proportion of adolescents aged 15 to 19 is 9.3% of the city’s population, and school enrollment rates are high. The prevalence of excess weight in Peruvian adolescents is 14.2%, and prevalence has not declined in recent years. Also recently, NCDs and their risk factors have gained more attention in public health and policy areas, with regulatory action focusing on healthful nutrition to address obesity and related NCDs. The *Multiplicadores Jóvenes* (Young Multipliers) project was conducted among adolescents aged 15 to 17 from 9 public secondary schools in peri-urban areas of Lima, Peru.

**Methods:**

The project provided basic communication tools and knowledge of NCD prevention and public health research to adolescents during 16 weekly participatory sessions to enable them to design and disseminate healthful lifestyle promotion messages to their school peers.

**Outcome:**

Thirty of 45 participants finished the program. Seven communications campaigns were designed and implemented in schools, reaching 1,200 students. The participants gained motivation, increased knowledge, and developed communication skills that were combined to implement healthful lifestyle promotion campaigns.

**Interpretation:**

Engaging young people in public health promotion activities was feasible and advantageous for the design of tailored prevention-related content and its dissemination among peers.

## Background

In 2010, 65% of deaths worldwide were attributed to noncommunicable diseases (NCDs) (1); the increasing prevalence of NCDs among adolescents is a significant public health problem (2,3). Many risk factors for NCDs among adults are associated with behaviors, such as poor dietary habits and physical inactivity, learned during childhood and adolescence (4). Targeting young people for NCD prevention initiatives may improve long-term outcomes and lead to reductions in rates of adult obesity (4). Early intervention is also important because of the long-term development of chronic conditions and their long duration once they are established.

Plasticity and adaptability are fundamental strengths of adolescence (5), so this life stage is an ideal time to intervene. The promotion of healthful habits such as physical activity and good nutrition during adolescence could have both immediate and long-term effects on public health. Given the right tools and contextual resources, adolescents can develop lifestyle habits that will last throughout life (6).

Engaging the community in public health research may enhance the community’s ability to address its own health needs and reduce disparities while ensuring that researchers understand community priorities (7). Involving young people as partners in the design and implementation of NCD prevention activities is a novel and potentially powerful approach to improving health knowledge and behaviors among young people. They can influence their peers by using their own codes, languages, and communication channels, thereby enhancing the impact of the prevention message (8,9).

The *Multiplicadores Jóvenes* (Young Multipliers) project was designed to provide communication tools, information, and support to adolescents in Lima, Peru, to actively engage them in the development of their own messages for peer promotion of healthful lifestyle behaviors.

## Community Context

### Setting and population

In 2013, 9 million of 30.5 million Peruvians lived in Lima, Peru’s capital city. One-third of Lima’s inhabitants are within-country immigrants, making Lima a culturally diverse environment with major socioeconomic disparities (10). This project was conducted in 3 geographic areas of Lima, Ate (east), Independencia (north), and Villa El Salvador (south), all of them in peripheral areas of the capital that historically have served as receiving areas for immigrants. In Lima, 1 in 3 people are aged 19 or younger, and 9.3% are aged 15 to 19 (10); the enrollment rate in secondary school and high school is 94% (11).

### Burden of NCDs and main risk factors

Low- and middle-income countries are undergoing a shift from infectious and communicable diseases to chronic diseases and experiencing the economic impact of NCDs (12). Peru is a middle-income country where NCDs are responsible for 66% of total deaths (13). During the last decade, the mortality pattern has rapidly changed from one dominated by infectious diseases to one dominated by NCDs and injuries (14). NCD risk factors are strongly associated with lower socioeconomic status in Peru (15).

Most data available for NCDs and their main risk factors among Peruvian children and adolescents are related to overnutrition. Rates of overweight and obesity among Peruvian children and adolescents are high (16) and have not declined in the past 15 years (17). For instance, in 2010, rates of excess body weight, including overweight and obesity, were 24.4% among children aged 5 to 9 years and 14.2% among those aged 10 to 19 (16). These estimates of excess body weight are similar to regional Latin American rates: 18.9% to 34.5% among children aged 5 to 11 years and 16.6% to 35% among those aged 12 to 19 (18).

### Schools as a target for health promotion

Because the school enrollment rate is so high in Lima, schools are an ideal place to explore and develop prevention strategies. Schools, however, can also serve as environments that promote unhealthful lifestyles — for example, by offering processed foods and beverages — and, conversely, that discourage healthful lifestyles — for example, by limiting opportunities for active movement and reducing time allocated to exercise (19). A study of nearly 1,800 school children in 80 primary schools in Lima found that half of the children had low physical activity levels (20). In alignment with other Latin American countries (21,22), obesity and NCD prevention is a priority for the Peruvian government; for example, a recent law restricts the marketing of processed foods to children and adolescents in school environments (23).

### Project objectives

Our project aimed to train a group of Peruvian adolescents (aged 15 to 17), named *multiplicadores jóvenes* (MJs), to design and disseminate messages promoting healthful lifestyles among their school peers. Our global objective was to engage adolescents in the community in playing an active role in health promotion by multiplying their messages to raise awareness about NCDs and unhealthful lifestyles among other young Peruvians.

## Methods

### Schools and students selection

Our project was conducted from January through December 2011. Nine public schools were chosen from the 3 geographic areas: 3 from the south, 3 from the north, and 3 from the east of Lima. In Peru, public schools are government funded and are the main providers of education services.

The following criteria were used for selecting schools: public, coeducational, at least 200 students per school, students studying in morning shift, and schools close to each other (maximum 10 minutes by public transportation). The decision to choose schools according to distance was to enable after-school group activities among students from different schools.

Selection of students was conducted with the support from tutors of each school. Tutors are members of the school’s staff who, in addition to their teaching responsibilities, are also assigned to look after a whole grade. Because of their roles, tutors spend more time with students during school hours and usually are more acquainted with them. Tutors were asked to select students based on leadership, motivation toward the project, and availability. We did not establish a quota according to sex. For each school, we requested tutors to select 5 students in the 4th or 5th secondary grades (in a 5-year secondary school plan). The rationale for selecting final-year students was based on the students’ potential to understand technical knowledge, their ability to use the project’s communications tools, and their potential influence as role models for younger peers. Our team held a 30-minute meeting with the students being recruited, where we described the project in detail and clarified expectations of both the students and the research team, so that a true commitment to the project was generated. We initially recruited 45 students: 33 girls and 12 boys.

### Training sessions

The 16 training sessions (Box) were led by 2 young professionals in social communications. Almost every week, from April through September, MJs attended training sessions divided into 3 groups, 1 group for each geographic area (north, south, and east). These sessions were explanatory, participatory, and dynamic, and emphasized straightforward language and group work. Social networking (ie, Facebook) was an important tool for communication and integration within student groups.

**Box. Training Sessions Schedule for the *Multiplicadores Jóvenes* (MJs) Program**

**Table:** 

Session	Type of session	Content/Topic
Session 1	Workshop 1	Presentation of project coordinators to MJs; communication and public health
Session 2	Informative session 1	Presentation of research team to MJs; noncommunicable diseases: basic concepts, risk factors, consequences
Session 3	Workshop 2	Communication: target audience and messages
Session 4	Experiential session 1	Clinical hypertension; hands-on activity: weight and height measurement, calculation of body mass index
Session 5	Workshop 3	Building a message: video format
Session 6	Informative session 2	Investigation: basic principles and process of investigation
Session 7	Workshop 4	Building a message: radio and graphic design formats
Session 8	Workshop 5	Communications campaigns: basic principles
Session 9	Workshop 6	How to design a health communications campaign
Session 10	Experiential session 2	Visit to research site, community fieldwork; hands-on activity: blood pressure measurement
Session 11	Workshop 7	Designing a proposal of a health communications campaign
Session 12	Feedback session 1	Research team and MJs discussion about proposals for communications campaigns
Session 13	Workshop 8	Production of MJs campaigns (videos, radio spots, graphic designs)
Session 14	Workshop 9	Production of MJs campaigns (videos, radio spots, graphic designs)
Session 15	Workshop 10	Production of MJs campaigns (videos, radio spots, graphic designs)
Session 16	Feedback session 2	Presentation of MJs campaigns

Training sessions were classified into 4 categories: workshops, experiential sessions, informative sessions, and feedback sessions. The workshops and experiential sessions were conducted separately for each of the 3 geographic areas to ensure working in small teams. In contrast, informative and feedback sessions, which required more specialized professionals, were conducted as a single activity with all participants from all schools together.

We started with a workshop to provide a solid background on communication skills. Feedback sessions took place during the last 2 months of the project, after MJs had their communication training. Workshops and experiential and informative sessions took place throughout the project.

**Figure 1:**
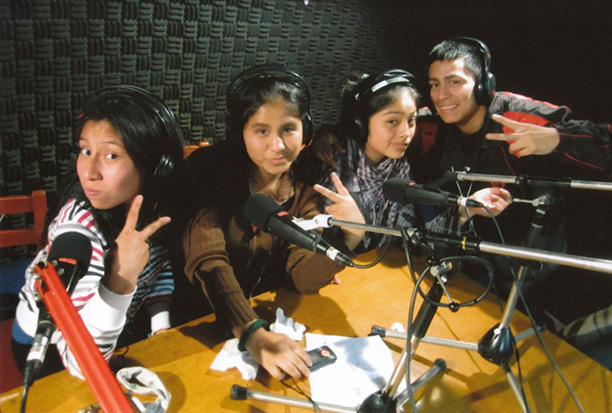
*Multiplicadores Jóvenes* visiting a radio production facility. The project enabled the contact with communication professionals and tools. Here, some students recorded their voices for a video used for their campaign.

**Workshops.** Based on their professional and teaching experience, the social communications professionals developed and led 10 workshops in which MJs were introduced to methods of communication through interactive and participatory exercises. Simple tools (eg, communication theory, human language, verbal and nonverbal language) and technologies (eg, blogs, social networks) were introduced to the MJs to help them develop effective messages and communication products for young audiences (Figure 1).

**Experiential sessions.** In 2 sessions, MJs worked with local practitioners, 1 physician trained in internal medicine and 1 physician trained in public health, who introduced them to public health issues and basic principles of clinical and public health research. MJs took part in public health fieldwork research by participating in community field visits and taking blood pressure and body and weight measurements among themselves.

**Informative sessions.** Researchers from our team led these 2 sessions. The first session aimed to build understanding of NCDs in lay terms and focused on diabetes and cardiovascular diseases and its main risk factors (eg, physical inactivity, junk food consumption, high salt intake). The aim of the second session was to describe the basic principles, the process, and the relevance of public health research.

**Feedback sessions.** In these 2 sessions, MJs groups presented a detailed description of their campaigns and received feedback from a panel of experts including professionals from communications, clinical medicine, and health research.

To guarantee participation in all of these activities, transportation was provided or its cost was reimbursed to MJs if needed. To concur with the spirit of the initiative, healthful food snacks were provided during the sessions. Also, to encourage engagement among all MJs, 2 local trips were organized to public spaces in Lima, and the project team covered transportation costs and entrance fees.

### Communications campaigns

During the last 4 workshops, each MJ team developed a health promotion communications campaign. MJs were free to choose the topic, target audience, and campaign strategy. Students were asked to use the approaches they believed would be the best for their target audiences. MJs contacted the project’s clinical practitioner to verify health-related content when necessary.

For production, each team was allocated a small amount of funds to secure materials and services (eg, rental of equipment or recording rooms). In addition, each school team was mentored by a communications professional to support final production and execution. No compensation was offered to MJs. A single prize, an MP3 player, was given to the MJ group with the best campaign.

### Project team

Two young social communicators were hired part-time to coordinate the process of communicating with schools, selecting students, and developing the training sessions. These communicators were supervised by a researcher from our group with a background in psychology and public health. After the training sessions, in preparation for the final presentation of their campaigns, MJs received onsite support from additional communications professionals. The role of these professionals, hired part-time for 2 weeks, was to refine and fine-tune each campaign.

### Evaluation

We evaluated our project in 3 areas. First, we monitored attendance of MJs at training sessions. Second, communications campaigns were considered successful if they were developed and delivered by MJs to their school peers. In addition, an independent evaluator conducted focus groups with 23 MJs from 7 schools after the completion of the campaigns to obtain information on the perceptions among MJs about the project.

This study was approved by the institutional review board at Universidad Peruana Cayetano Heredia, Lima, Peru; each MJ assented to participate, and parents consented to their participation.

## Outcome

### Training sessions

Thirty of 45 students completed the training; of those who dropped out, 9 students were from 2 schools. During the focus groups, several MJs described their participation as a great life experience. According to them, the most valuable aspect of the training was learning about healthful lifestyles and NCDs, which motivated them to improve their own eating habits and to persuade their peers to avoid unhealthful behaviors such as consumption of junk food: “I learned a lot about NCDs. . . . Now, I take more care of myself. . . . I do not buy much junk food from the school kiosk . . . but I do not drink enough water . . . that is something I still need to change.” In addition, MJs related their learning to their situation at home: “[O]ne of my grandparents suffers from diabetes and the other has hypertension. Now I know better how to take care of them.”

Most MJs positively rated the playful atmosphere and straightforward language of the sessions, the quality of educational videos, the visits to our Center’s research facilities, and the opportunity to meet students from other schools and learn about teamwork. Another aspect valued by MJs was the attention paid to details: “[T]he lunchboxes that we received during prolonged training sessions had fresh juice, and fruits — no sodas or potato chips!”

### Communications campaign implementation

**Figure 2:**
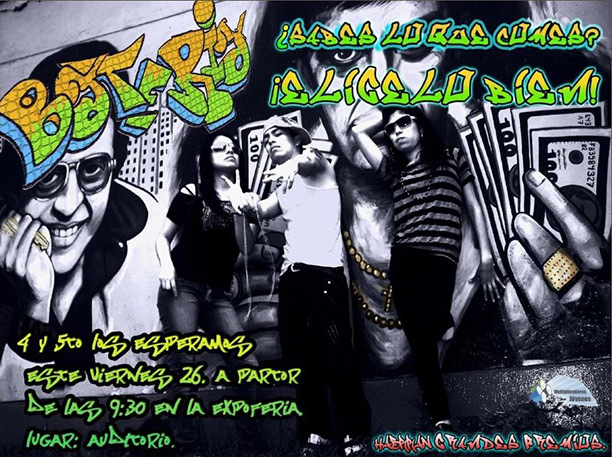
Poster produced by *Multiplicadores Jóvenes* as part of a school-based communications campaign. The advertisement reads “*Batería, ¿sabes lo que comes? ¡Elígelo bien!*” (“Dude, do you know what you’re eating? Choose well!”).

The communications campaigns ran in 7 of 9 schools, reaching about 1,200 students. Target audiences were reached through innovative strategies: video reports, video clips, radio spots, graphic design advertisements, and flash mobs (a short entertaining activity performed in a public place). All campaigns featured the promotion of healthful food and rejection of junk food and sodas (Figure 2) because MJs considered these topics to be most relevant to their peers and themselves: “[W]e focused on junk food, and our goal was for young people to get our message about which foods are good and bad, because we wanted to spread the information we learned in the training session to others.”

All MJs greatly appreciated the experience of running their own campaigns, an activity that they considered the most important of the project. The organization and execution of the campaigns, not always supported by schools authorities, demanded great effort and time from the MJ teams, especially because of their small size, and was also an opportunity to overcome personal fears and discover communication and leadership skills: “I learned how to speak in public, I used to be afraid of it but I lost that fear. . . . I never thought I would record a radio spot, design a campaign, be part of a working group.”

Dynamic hands-on methods and staff commitment to the project encouraged MJs to increase their own involvement: “[O]n the central campaign day, the project promoter arrived at school at 7 am, right when we arrived: their sacrifice was similar to ours.”

Most MJs believed their peers were receptive to the campaign messages and showed commitment to the campaign activities. After the project, some MJs felt that they had more recognition not only from their peers but also from school’s teachers and were motivated to be part of similar initiatives in the future.

### Difficulties

Fifteen students, 9 of whom were from the 2 schools that did not implement a campaign, discontinued the project. The main reason for discontinuing was a heavy workload in addition to school duties. Several participants had education-related activities after school hours, and others had to take care of relatives or work to support their household. In most schools, tutors and authorities were not interested in collaboration, and there was little flexibility with timetables to allow MJs to participate.

At the beginning of the project, some parents had concerns about project activities that took place outside school hours. These concerns not only cast doubts on the relevance of the project but also signaled conflicting demands related to employment and money issues.

Sometimes, the workload required to design and implement the project simultaneously in 9 schools overcame the capacity of our part-time coordinators. Similarly, the number of MJs selected per school, up to 5, was small, considering the amount of time involved in developing and implementing a campaign.

## Interpretation

The concept of engaging young MJs on topics of public health promotion and prevention was effective, given that a group of adolescents gained motivation, basic scientific and public health knowledge, and media skills during the project. They implemented communications campaigns in their schools to convey to their peers what they had learned through culturally and age-relevant communication strategies (http://youtu.be/I3Aq3QswkvY).

Because campaigns took place during a short time, they may have reached their target audience only superficially. Despite the short time, this type of engagement demonstrated the feasibility of engaging young people in developing and delivering prevention messages. Training sessions could be repeated in Lima and in similar Latin American contexts, particularly in intensive research field sites.

This project also provided lessons and insights for the future. The process of selecting MJs could be improved by 1) identifying a better balance between commitment and availability to accommodate the demands of the project and the school and after-school routines of the students, 2) pursuing a more proactive balance between girls and boys, 3) convening a greater number of MJs per school, including younger MJs, and 4) exploring whether peers could take part in the selection process as tested elsewhere (24). Some of these elements could increase student participation, reduce nonattendance rates, and improve sustainability.

Other suggestions are to consider providing incentives for student participation and to incorporate training sessions into standard school hours. It would be useful to establish selection criteria for schools and to define clearly the responsibilities of the school during the implementation phases. Providing incentives to encourage collaboration of schools and their higher authorities might also help. Communication with parents to address their concerns and encouraging them to participate with their children would help secure the commitment of participants and their families. If resources are available, it would also be desirable to include standardized measurements of impact during the training and campaign phases. To ensure sustainability, scale-up, and a broader impact of the project, we should aim to integrate our program with larger public institutions in Peru, such as the Ministry of Health, Ministry of Education, or other local authorities.

This project focused on providing communication tools, information, and support to young people to actively engage them in the development of their own messages for peer-to-peer promotion of healthful lifestyle behaviors. Innovative, pragmatic, and effective ways to communicate with peers and other audiences were developed. This communication could contribute to wider health promotion and potential public health gains given that NCD prevention requires reaching people early in life. In addition, by improving accessibility to researchers, people could become encouraged to participate more actively in research, which may also be beneficial for NCD prevention. This project provided an ideal opportunity for and demonstrated the feasibility of bringing together researchers, communicators, and adolescents.
